# Barriers towards effective pharmacovigilance systems of biosimilars in rheumatology: A Latin American survey

**DOI:** 10.1002/pds.4785

**Published:** 2019-05-30

**Authors:** Gilberto Castañeda‐Hernández, Hugo Sandoval, Javier Coindreau, Luis Felipe Rodriguez‐Davison, Carlos Pineda

**Affiliations:** ^1^ Departamento de Farmacología Centro de Investigación y de Estudios Avanzados del Instituto Politécnico Nacional Mexico City Mexico; ^2^ Sociomedical Research Unit Instituto Nacional de Rehabilitación Luis Guillermo Ibarra Ibarra Mexico City Mexico; ^3^ Pfizer Inc New York NY USA; ^4^ Pfizer Biosimilars Latin America Mexico City Mexico; ^5^ Division of Musculoskeletal and Rheumatic Diseases Instituto Nacional de Rehabilitación Luis Guillermo Ibarra Ibarra Mexico City Mexico

**Keywords:** biosimilars, Latin America, pharmacoepidemiology, pharmacovigilance, rheumatology

## Abstract

**Purpose:**

This review summarises the current status of regulatory guidelines for the approval of biosimilars in Latin America and highlights the main barriers to effective pharmacovigilance in this region. We also report results from a survey of Latin American rheumatologists assessing their understanding of prescribing biosimilars and the pharmacovigilance of these drugs.

**Methods:**

We reviewed the current guidelines for the regulatory approval of biosimilars and barriers to effective pharmacovigilance in Latin American countries. Rheumatologists attending the II Pan‐American League of Rheumatology Associations PANLAR Review Course (Biosimilars update) in Lima, Peru were asked to complete a short survey to determine their knowledge of biosimilars.

**Results:**

Many Latin American countries continue to lag behind Europe and the United States in establishing regulatory guidance and effective pharmacovigilance systems for biosimilars. Results from our survey also highlight a lack of awareness regarding the availability of biosimilars, their nomenclature, automatic substitution, and reporting adverse drug reactions because of these drugs.

**Conclusions:**

The main barriers to effective pharmacovigilance in Latin America are the lack of consensus on the interchangeability of reference biologics and biosimilars, and the need for more suitably trained personnel to carry out effective postmarketing pharmacovigilance of biosimilars. Inconsistencies in biosimilar nomenclature make it difficult to adequately trace drugs and record adverse drug reactions associated with their use, creating a barrier to the global pharmacovigilance of biologics.

**Table 1:** Nomenclature and definitions used for biosimilars and noncomparable biotherapeutics

Term	Definition	Reference
Biosimilar	A biopharmaceutical that is highly similar to an already licensed biologic product (the reference product), notwithstanding minor differences in clinically inactive components, and for which there are no clinically meaningful differences in purity, potency, and safety between the two products	FDA^5^
	A biological medicinal product that contains a version of the active substance of an already authorised original biological medicinal product (reference medicinal product). Similarity to the reference medicinal product in terms of quality characteristics, biological activity, safety, and efficacy, based on a comprehensive comparability exercise, needs to be established	EMA^6^
Noncomparable biotherapeutic	Biotherapeutic medicinal products that are intended to “copy” another biotherapeutic product; have not been directly compared and analysed against an already licensed reference biotherapeutic product; and have not been approved via a regulatory pathway that is in alignment with World Health Organization Similar Biotherapeutic Product guidelines that ensure quality, safety, and efficacy	IFPMA^7^
Interchangeability	One medicine is exchanged for another medicine that is expected to have the same clinical effect. For example, a reference product could be replaced with a biosimilar (or vice versa), or one biosimilar could be replaced with another	EMA^8^
	A biosimilar is designated as interchangeable if it is “expected to produce the same clinical result as the reference product in any given patient” and if a biological product “is administered more than once to an individual, the risk in terms of safety or diminished efficacy of alternating or switching between the biological product and reference product is not greater than the risk of using the reference product without such alternation or switch”	FDA^9^
Switching	The prescriber decides to exchange one medicine for another medicine with the same therapeutic intent	EMA^8^
Substitution (automatic)	The practice of dispensing one medicine instead of another equivalent and interchangeable medicine at pharmacy level without consulting the prescriber	EMA^8^
Extrapolation	The approval of a biosimilar for use in an indication held by the reference product but not directly studied in a comparative clinical trial with a biosimilar	Tesser, Furst, and Jacobs^10^

Abbreviations: EMA, European Medicines Agency; FDA, US Food and Drug Administration; IFPMA, International Federation of Pharmaceutical Manufacturers and Associations.

**KEY POINTS**

- Many Latin American countries lag behind Europe and the United States in establishing regulatory guidelines and effective pharmacovigilance systems for biosimilars.
- Inconsistencies in the nomenclature of biosimilars make it difficult to adequately trace drugs and record adverse drug reactions, creating a barrier to global pharmacovigilance.
- For effective pharmacovigilance input from academic bodies and regulatory agencies, it is vital to agree a common definition and legislation on the interchangeability of reference products and biosimilars.
- More suitably trained personnel are needed to carry out effective pharmacovigilance of biosimilars in Latin American countries.

## 1 INTRODUCTION

As patent portfolios for reference biologics near end of term, pharmaceutical companies are developing safe and effective biosimilars for these drugs.^1^, ^2^, ^3^, ^4^ A biosimilar is highly similar to an approved reference product, with no clinically meaningful differences in purity, potency, and safety (Table 1).^5^, ^6^, ^7^, ^8^, ^9^, ^10^ Biosimilars can potentially increase patient access to more affordable biologic treatments and have an important role in the treatment of chronic conditions, including rheumatic and musculoskeletal diseases.^11^, ^12^ Several biosimilars are approved for treating patients with rheumatic and musculoskeletal diseases such as rheumatoid arthritis, ankylosing spondylitis, idiopathic juvenile arthritis, psoriatic arthritis, and other immune‐mediated inflammatory conditions.^2^, ^13^ In addition, several potential biosimilars are in development.

As with reference biologics, biosimilars can cause an immunogenic response in treated individuals. The immune system can induce the development of antidrug antibodies in response to a biologic, which can impact the medicine's clinical efficacy and increase the risk of adverse drug reactions (ADRs).^14^ Adverse events (AEs) such as cardiotoxicity, cytokine‐release syndrome, and reactivation of latent tuberculosis can also be encountered with biologic treatments.^15^ To ensure patient safety, it is important to monitor immunogenicity and ADRs during drug development and through postmarketing surveillance to gain real‐world clinical experience.^16^, ^17^ Effective pharmacovigilance systems are essential for detecting, reporting, understanding, and preventing ADRs. Unfortunately, only a few Latin American countries have the necessary systems in place to collect, manage, and analyse reported safety data.^18^

Pharmacovigilance is also important for monitoring biologic use in specific populations, as the efficacy and safety of these drugs vary among different ethnic groups. For instance, in Japanese patients with rheumatoid arthritis, higher clinical response rates have been observed with biologics—including infliximab, etanercept, and tocilizumab—compared with patients from Western countries.^19^ Moreover, a lower incidence of peri‐infusional ADRs to rituximab was reported in Mexican patients compared with studies in other ethnic groups.^20^ Given the demographic heterogeneity of some Latin American populations, the variation in pharmacogenetic biomarkers in these patients should be a consideration for the pharmacovigilance of biologics.^21^

Pharmacovigilance systems are well established in Europe and the United States. However, most Latin American countries lag behind developed countries in establishing regulatory guidance and effective pharmacovigilance for biosimilars.^18^ In many Latin American countries, pharmacovigilance systems are suboptimal; so healthcare professionals in these regions play a key role in postmarketing surveillance.^17^, ^22^ There are two main barriers to effective pharmacovigilance in Latin America: first, the lack of consensus on the interchangeability of reference products and biosimilars^7^, ^17^; second, the need for more suitably trained personnel to carry out postmarketing surveillance of biosimilars.^17^ To facilitate effective pharmacovigilance, it is important to trace reference biologics and biosimilars after their approval for use in clinical practice, to ensure accurate reporting of ADRs.

This review highlights the status of regulatory guidelines for the approval of biosimilars in Latin America compared with the rest of the world and describes the main barriers to effective pharmacovigilance. We also conducted a survey to gain an insight into the heterogeneous position on biosimilars between Latin American countries regarding topics such as the pharmacovigilance and regulation of these drugs. We report results from this survey assessing current awareness of these key issues among Latin American rheumatologists.

## 2 REGULATORY APPROVAL OF BIOSIMILARS: GLOBAL VERSUS LATIN AMERICAN GUIDELINES

The assessment of similarity involves iterative structural and functional characterisation and, if needed, in vivo non‐clinical evaluation and clinical studies, all comparing the potential biosimilar with its reference product.^1^ However, there are disparities across the world in the regulatory approval pathways for biosimilars, and on the interchangeability of reference products and biosimilars at the prescriber or the pharmacy level.^18^, ^23^, ^24^

### 2.1 European Union

The European Medicines Agency (EMA) guidelines for approval of biosimilars are well established; the first biosimilar was approved in the European Union (EU) in 2006.^8^, ^25^ However, guidance regarding interchangeability, switching, and substitution of a reference product with a biosimilar is determined by individual EU member states.^8^ To date, no EU‐approved biosimilars have been withdrawn or suspended because of safety concerns.^26^

### 2.2 United States

The United States Food and Drug Administration (FDA) has published guidelines for the approval of biosimilars and first approved a biosimilar in 2015.^5^, ^27^ In contrast to EMA guidelines, the FDA can designate a biosimilar as “interchangeable”, although individual states also regulate interchangeability.^28^ When interchangeability status is granted, a biosimilar can be substituted for its reference product at the pharmacy level without further input from the prescriber. However, interchangeability is not automatically granted upon approval of a biosimilar; additional evidence is required to demonstrate that the clinical result achieved with the biosimilar is expected to be the same as the reference product. In addition, switching treatment between the reference product and biosimilar should demonstrate no increased risk to the patient.^9^, ^29^

### 2.3 Rest of the world and the World Health Organization

In 2009, World Health Organization (WHO) published guidance documents, based on those from the EMA, with the aim of providing “globally acceptable principles for licensing products … claimed to be similar to licensed biotherapeutic products of assured quality, safety, and efficacy that have been licensed based on a full licensing dossier”.^30^ Many other countries have established or are developing guidelines for the approval of biosimilars, based on EMA and WHO guidance.^28^

### 2.4 Latin America

Some Latin American countries have published guidelines for biosimilar approval, based on EMA and WHO guidance, while others have issued only draft documents or none at all (Table 2).^17^, ^18^, ^31^ Brazil has two regulatory pathways for biosimilars—a “comparative pathway” and an “individual development pathway”.^18^, ^32^ The former requires preclinical and clinical data to demonstrate similarity to the reference product; only products approved via this pathway are considered to be biosimilars. In contrast, the individual development pathway does not compare the potential biosimilar with the reference product; rather, summaries of preclinical and clinical studies are required.^32^, ^33^

**Table 2:** Status of guidelines on biosimilar approval in Latin American countries^17^, ^18^, ^31^

Status	Country	Year of guideline publication, and regulatory agency
Published guidelines	Argentina	2011 Administración Nacional de Medicamentos, Alimentos y Tecnología Médica (ANMAT)
	Brazil	2010 Agência Nacional de Vigilância Sanitária (ANVISA)
	Chile	2014 Agencia Nacional de Medicamentos (ANAMED)
	Colombia	2014 Instituto Nacional de Vigilancia de Medicamentos y Alimentos (INVIMA)
	Costa Rica	2012 Ministerio de Salud
	Cuba	2011 Centro para el Control Estatal de Medicamentos, Equipos y Dispositivos Médicos (CECMED)
	Dominican Republic	2016 Ministerio de Salud Pública
	Guatemala	2010 Ministerio de Salud Pública y Asistencia Social (MSPAS)
	Mexico	2014 Comisión Federal para la Protección contra Riesgos Sanitarios (COFEPRIS)
	Panama	2007 Ministerio de Salud Panama
	Paraguay	2015 Ministerio de Salud Paraguay
	Peru	2012 Ministerio de Salud Peru
	Uruguay	2015 Registro de Medicamentos Biotechnologicos
	Venezuela	2012 Ministerio del Poder Popular para la Salud (MPPS)
Draft guidelines in development	Bolivia	Not published
No specific guidelines in place	Ecuador	‐

Colombia has three regulatory pathways for biosimilars; the “complete dossier” approach, the “comparability approach”, and an “abbreviated comparability approach”.^34^ These pathways share common elements, and the pathway followed will depend on the biologic submitted for approval. For example, the “abbreviated comparability approach” is followed when the reference product is sufficiently characterised, with well‐defined safety and efficacy, and adequate data are available in terms of clinical experience and pharmacovigilance evidence.^34^

In Mexico, biologics (including biosimilars) have been available for a number of years.^35^ Biologics were included in the Mexican General Health Law in 2009; this law was amended in 2011 and establishes the requirements for approval of biologics including biosimilars (known as biocomparables in Mexico).^36^ There is now an Official Mexican Standard for all biologics, including biosimilars, that came into effect in 2014, which provides guidance on generating clinical protocols, quality management systems, pharmacovigilance, and demonstrating biosimilarity to reference products.^36^

## 3 PHARMACOVIGILANCE OF BIOSIMILARS IN LATIN AMERICA

### 3.1 Nomenclature for biologics and biosimilars

One topic still under debate is biosimilar nomenclature. In Europe, biosimilars have distinct brand names, whereas the FDA's naming convention combines the non‐proprietary name with a suffix of four lower‐case letters (devoid of meaning).^37^ However, regulations in Latin American regions do not require different nomenclature for a biosimilar and reference products.^18^ For example, in Colombia and Mexico, physicians prescribe drugs using the international non‐proprietary names (INNs) and specific codes established by the social security system, while in Brazil, trade names are not used on prescriptions.^17^, ^18^, ^38^ This makes it difficult to adequately trace drugs and record any ADRs associated with their use, creating a barrier to global pharmacovigilance. For example, in a Mexican pharmacovigilance study on filgrastim, four products—the reference product and three noncomparable biotherapeutics (intended copies)—were dispensed simultaneously using the same code number. As such, it was not possible to identify the filgrastim brand that each patient received or to trace ADRs associated with each drug using the INN alone.^38^

Therefore, to ensure ADRs are accurately reported, a unique identifier is needed to distinguish biosimilars and noncomparable biotherapeutics from the reference product.^31^ WHO proposed the use of “biological qualifiers” (four random consonants and an optional two digits) for all biological active substances with an INN, to ensure a consistent naming approach. This code, together with the INN, would allow the biologic's manufacturer and country of origin to be traced, which could improve global pharmacovigilance.^39^ These proposals are currently on hold as no consensus has been reached on the use of biological qualifiers.^40^

### 3.2 Biologic registries: Monitoring treatment efficacy and safety

Several European countries have well‐established biologic registries that facilitate accurate information regarding treatment and ADRs to be recorded for biologics used in rheumatology.^3^, ^41^, ^42^, ^43^ In 2007, several Latin American countries set up registries to monitor biologic use in their home countries, supported by the Pan‐American League of Rheumatology Associations (PANLAR).^44^ This collaboration, BIOBADAMERICA, comprised 15 countries and used the Spanish Society of Rheumatology registry (BIOBADASER) as a model.^45^ Each registry is owned by a national rheumatology society or association and has its own staff and governance.^44^, ^46^ Registries established in Brazil (BiobadaBrasil), Argentina (BIOBADASAR), and Mexico (BIOBADAMEX) have provided useful information on biologic use in clinical practice.^47^, ^48^, ^49^ However, substantial funding is required to implement and maintain these registries, resulting in inconsistent participation among countries.^3^, ^44^, ^46^

### 3.3 Use of noncomparable biotherapeutics, counterfeit, and stolen medicines in Latin America

The use of noncomparable biotherapeutics in Latin America presents a challenge for pharmacovigilance. These drugs are copies of reference biologics introduced before the release of regulatory guidance. As a result, they have not met the requirements for establishing biosimilarity to the reference product (Table 1).^7^ Without suitable pharmacovigilance systems in place, it is difficult to establish the potential risk of these agents in clinical practice. Experts have recommended that noncomparable biotherapeutics should be re‐evaluated using regulations for the approval of biosimilars.^31^, ^50^ This is now a requirement of Mexico's regulatory agency, the Federal Commission for the Protection Against Sanitary Risk.^50^

A further challenge for pharmacovigilance is the sale and use of counterfeit and stolen medicines. ADRs resulting from their use are not accurately recorded, which is a problem in regions lacking robust regulatory systems.^51^ For effective pharmacovigilance, it is imperative that biosimilars and noncomparable biotherapeutics can be traced after their approval for use in clinical practice.^17^ In Latin America, access to biosimilars is hampered by stock‐outs in public healthcare systems and high out‐of‐pocket expenditure on medicines.^52^ These factors encourage the use of stolen medicines and their resale on the black market. In addition to ensuring patient safety, traceability programmes could help deter prescription drugs being diverted from legitimate sources to illegal marketplaces during their delivery.^53^, ^54^

### 3.4 Pharmacovigilance and interchangeability: A traceability approach

The terms “interchangeability” and “substitution” should be differentiated from each other (Table 1). Interchangeability permits a prescriber to replace a reference product with a biosimilar, whereas substitution allows a biosimilar to be dispensed in place of a reference product without further input from the prescriber.^23^ Guidance on the interchangeability and substitution of biosimilars, either with reference products or another biosimilar, also differs between regulatory agencies.^18^, ^55^ This poses a challenge for postmarketing pharmacovigilance if automatic substitution is permitted without the original prescriber's input.^17^ Extrapolation of data across indications is not permitted in Brazil for products approved under the individual development pathway (see above).^32^, ^33^ The Brazilian National Health Surveillance Agency, ANVISA, considers interchangeability to be “more directly related to clinical practice than to regulatory status”. As such, the decision to switch treatment between biosimilars and their reference products should be made by the physician and patient. In addition, ANVISA does not recommend multiple switching between biosimilars and reference products, due to challenges relating to traceability.^56^, ^57^ The Mexican College of Rheumatology recommends that substitution of interchangeable biosimilars includes intervention from a healthcare professional.^58^

### 3.5 Establishing risk‐management plans for biosimilars

Risk‐management plans (RMPs) can assist with early pharmacovigilance planning for new drugs by assessing the potential risks of certain medicines and detailing how these issues will be addressed in postmarketing follow‐up.^17^ RMPs cover a medicine's entire life cycle and must be regularly updated as new safety information becomes available. The EMA requires an RMP to be submitted when applying for market authorisation of a biosimilar.^59^ The aim of these plans is to ensure that the benefits of using a particular medicine outweigh its risks.^9^, ^59^, ^60^ To improve pharmacovigilance in Latin America, RMPs should be essential for all biologics, including biosimilars.^17^ Mexico recently updated their official standard on pharmacovigilance, and RMPs are now mandatory.^61^

### 3.6 Reporting ADRs in Latin American countries: Key challenges

Healthcare professionals play a key part in improving pharmacovigilance through accurate reporting and recording of ADRs.^17^, ^22^, ^50^ In developing countries, healthcare is often fragmented, with limited financial resources for pharmacovigilance systems.^62^ There is also a lack of awareness among physicians about accurate reporting, which contributes to under‐reporting of ADRs.^63^, ^64^

To evaluate awareness of biosimilars and prescribing practices in Latin America, the Alliance for Safe Biologic Medicines conducted a survey among physicians in Argentina, Brazil, Colombia, and Mexico.^63^ The results indicated a lack of awareness about how biologics should be identified when reporting AEs. Around half of respondents stated they either rarely reported AEs or only reported some AEs for biologics. Prescribers under‐reported AEs largely because they were unsure of the reporting process and because of time constraints. In addition, the bureaucracy in distributing and returning completed reporting forms can be complicated.^62^ Another challenge in low‐income countries is that, as healthcare professionals have many patients to attend to, they may have insufficient time to complete the forms to report a suspected ADR.^62^

Given that ADRs are often under‐reported because of a lack of knowledge, there is a worldwide need for improved pharmacovigilance training for healthcare professionals, particularly in Latin America where few countries have the financial resources to invest in professional pharmacovigilance training.^62^, ^65^ There is also a need for training on biosimilars and the introduction of pharmacovigilance courses in medical schools in resource‐limited regions such as Latin America.^24^, ^62^, ^66^ The Brazilian Society of Clinical Oncology recommends that issues relating to pharmacovigilance should be addressed in medical meetings and congresses, and that biosimilar developers should be encouraged to have an active role in facilitating pharmacovigilance.^24^ Continued training using multifaceted educational interventions can facilitate accurate ADR reporting among healthcare professionals.^66^, ^67^, ^68^

Globalisation, innovative biotechnological drugs, and increased internet use can all influence the way people access information about medicines. These changes require countries and regulatory agencies to reassess their approach to pharmacovigilance. Involving patients in pharmacovigilance is important to minimise the risk associated with polypharmacy and to increase understanding of drug interactions (with food and other medicines) and nonconventional therapies. This will ensure that information about new ADRs is reported quickly and contributes to a better understanding of how drugs interact when used in real‐world settings.^69^

## 4 RHEUMATOLOGIST SURVEY: PERCEIVED BARRIERS TO PHARMACOVIGILANCE IN LATIN AMERICA

### 4.1 Design and methodology

**Figure 1:**
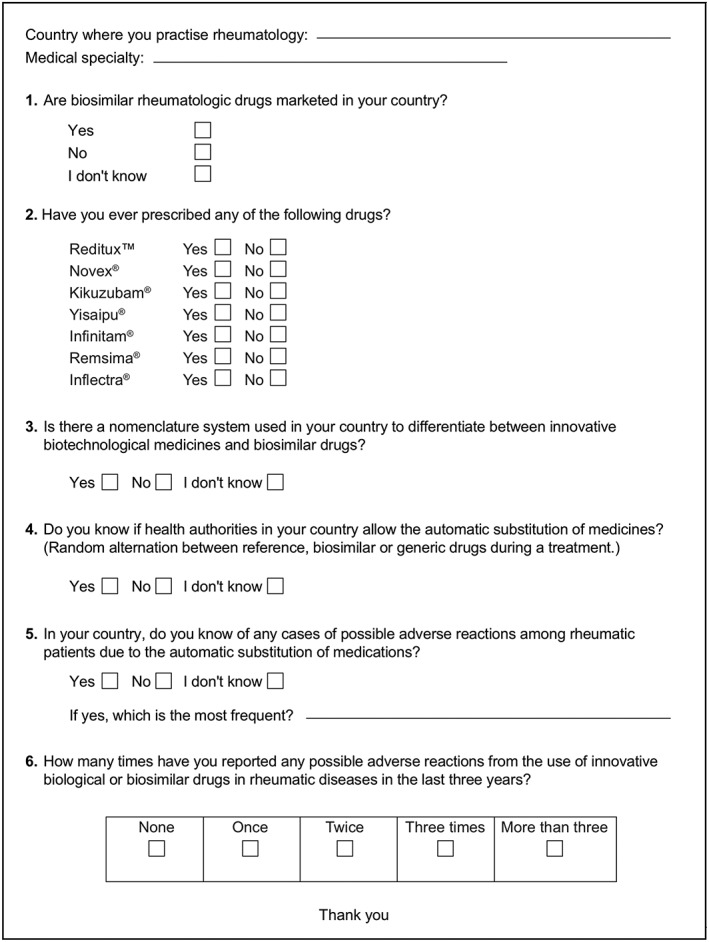
Survey for rheumatologists attending the II Pan‐American League of Rheumatology Associations (PANLAR) Review Course (Biosimilars update), in Lima, Peru (September 6‐8, 2017).^a,b^ *Note:* ^a^The survey was originally written in Spanish. ^b^Remsima and Inflectra are the product names for the infliximab biosimilar, CT‐P13, developed by Celltrion (Incheon, Republic of Korea) and marketed worldwide.^70^ Only Remsima is marketed in Latin America.^70^

Rheumatologists attending the II PANLAR Review Course (“Biosimilars update”) in Lima, Peru (6‐8 September 2017), were asked to voluntarily complete a short survey, comprising six questions (in Spanish) with multiple‐choice responses (Figure 1). The survey was designed to determine experts' awareness of biosimilars, including prescribing practices, nomenclature, automatic substitutions, and ADR reporting. The survey was conducted by administrative staff from PANLAR on behalf of G.C‐H., H.S., and C.P.

### 4.2 Results

In total, 104/155 (67%) rheumatologists completed the survey. Seven surveys were excluded from the analysis as they were incomplete.

### 4.3 Current awareness of biosimilars and prescribing practices

**Figure 2:**
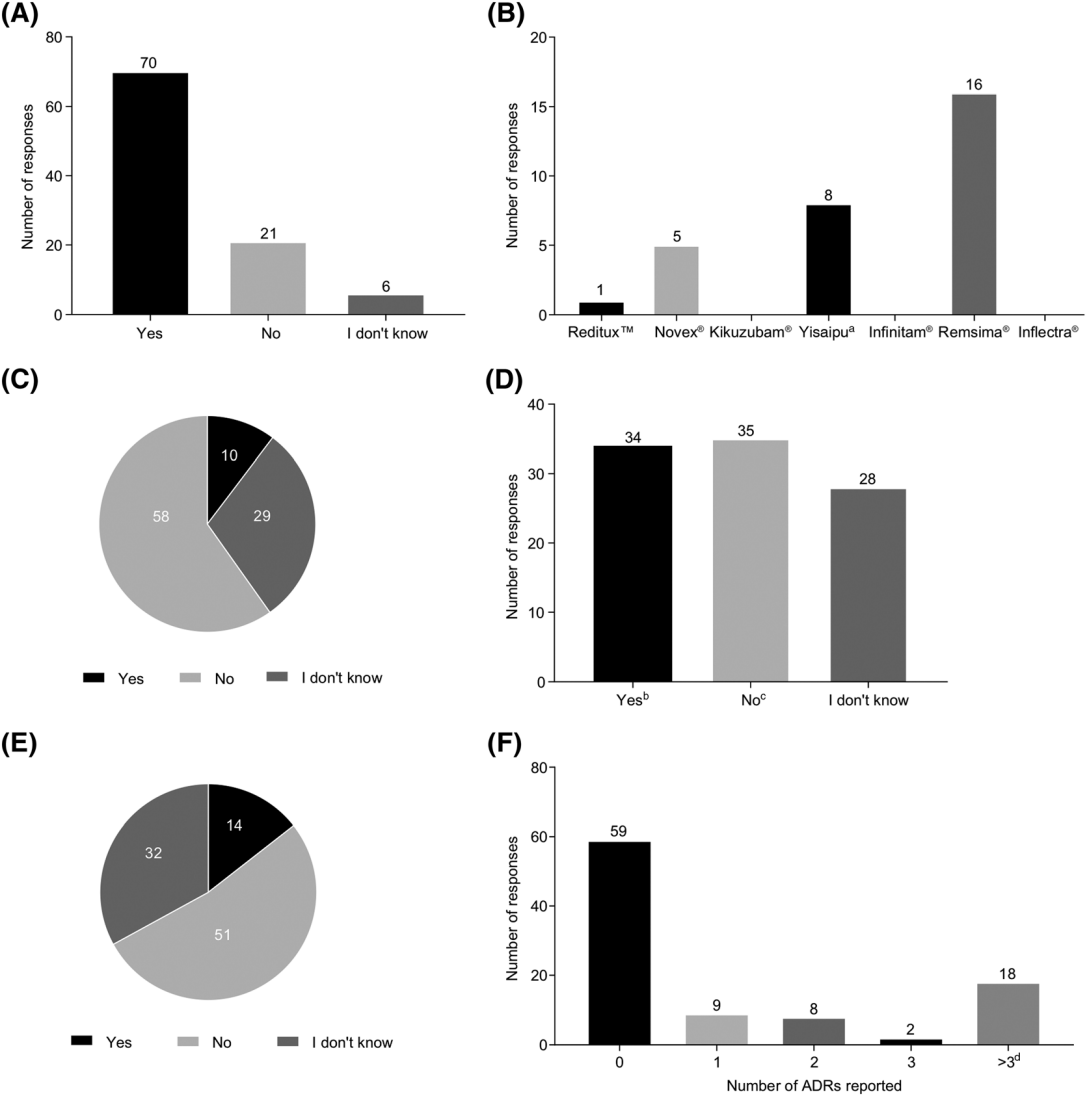
Awareness of prescribing practices for biosimilars and noncomparable biotherapeutics and pharmacovigilance among rheumatologists in Latin America. A, Awareness of biosimilars approved for use in rheumatology practice. B, Prescription of biosimilars and noncomparable biotherapeutics by rheumatologists. C, Awareness of the use of a nomenclature system for biologics, including biosimilars. D, Is automatic substitution of biosimilars and biologics permitted in the country where you practise rheumatology? E, Adverse drug reactions (ADRs) among rheumatic patients because of automatic substitution of biologics and biosimilars. F, Frequency of ADR reports due to treatment with biologics, including biosimilars, by rheumatologists during the past 3 years.^e^ Note: Responses were obtained from rheumatologists practising in Argentina, Bolivia, Brazil, Chile, Colombia, Costa Rica, Cuba, Dominican Republic, Ecuador, Guatemala, Honduras, Mexico, Nicaragua, Panama, Paraguay, Peru, United States, Uruguay, and Venezuela. ^a^Yisaipu is the Chinese brand name and is marketed as Etanar in Colombia and as Etart in Mexico.^13^ ^b^Rheumatologists from Argentina, Bolivia, Brazil, Chile, Colombia, Cuba, Ecuador, Mexico, Nicaragua, Peru, United States, Uruguay, and Venezuela. ^c^Rheumatologists from Argentina, Chile, Colombia, Costa Rica, Dominican Republic, Ecuador, Guatemala, Honduras, Panama, Paraguay, Peru, United States, and Venezuela. ^d^Rheumatologists from Argentina, Brazil, Colombia, Costa Rica, Dominican Republic, Panama, Peru, Paraguay, United States, Uruguay, and Venezuela. ^e^One rheumatologist did not provide a response for this question.

Most respondents indicated that biosimilars were available in the country where they practised (Figure 2A). However, some rheumatologists from Argentina, Chile, Peru, and Venezuela incorrectly reported that biosimilars were not approved for clinical use in these countries. Similarly, some rheumatologists from Bolivia and Peru were not aware of biosimilars or noncomparable biotherapeutics, even though they are approved in both countries. The only true biosimilar prescribed by the respondents was infliximab (Remsima) (Figure 2B). Remsima and Inflectra are the product names for the infliximab biosimilar, CT‐P13, developed by Celltrion (Incheon, Republic of Korea) and marketed worldwide.^70^ Only Remsima is marketed in Latin America.^70^ None of the rheumatologists reported the use of Inflectra. Noncomparable biotherapeutics for rituximab (Reditux and Novex) and etanercept (Yisaipu) were prescribed by rheumatologists from Venezuela, Argentina, and Colombia, respectively. However, none of the rheumatologists reported the use of noncomparable biotherapeutics for rituximab (Kikuzubam) or etanercept (Infinitam).

### 4.4 Nomenclature and automatic substitutions

The majority of respondents (58 rheumatologists) indicated that a naming system was not used to differentiate between reference products and biosimilars in the country where they practised, or they were not aware of such a system (29 rheumatologists) (Figure 2C). Approximately one third reported that automatic substitution between reference products and biosimilars was permitted in the country where they practised, while a further third reported that automatic substitution was not permitted (Figure 2D).

### 4.5 ADRs/pharmacovigilance notifications

Over half of the respondents (51 rheumatologists) were not aware of any ADRs because of automatic substitution of a reference biologic with a biosimilar (Figure 2E). In this survey, the most frequently reported ADRs resulting from substitution were anaphylactic reaction, joint pain, allergy, hypersensitivity, urticaria, and tachycardia. Most of the rheumatologists (59 respondents) had not reported possible ADRs due to biologics or biosimilars during the past 3 years (Figure 2F), while 18 respondents had reported more than three ADRs in the same period.

## 5 CONCLUSIONS

In Latin America, healthcare professionals play an important role in the pharmacovigilance of biologics, including biosimilars; however, they face several challenges. Key barriers to effective pharmacovigilance include the lack of international consensus on nomenclature to distinguish between reference biologics, biosimilars, and noncomparable biotherapeutics and the absence of clear guidance on interchangeability and substitution of a reference product with a biosimilar.^18^, ^71^ Although registries for monitoring biologics have been established in Latin America, their availability varies between countries, and there are also administrative barriers preventing healthcare professionals from reporting ADRs. It is important that suitable traceability strategies are established for accurately recording ADRs resulting from treatment with all biologics. In addition, pharmacovigilance training needs to be improved in Latin America so that sufficient resources and suitably trained personnel are available to perform pharmacovigilance studies.^17^, ^50^ Improved access to pharmacovigilance training in medical schools may reduce under‐reporting of ADRs related to biosimilar treatment, allowing an accurate assessment of their safety in clinical practice.

To address some of these barriers, rheumatology societies and biosimilar experts in Latin America have proposed several recommendations. One suggestion is to use a unique identifier throughout the region for noncomparable biotherapeutics and approved biosimilars. Another suggestion is to raise awareness about the importance of reporting ADRs resulting from treatment with biologics, including biosimilars, and to establish effective tracking systems to capture and analyse data.^50^ Physicians and regulatory agencies should work collaboratively to ensure the appropriate use of biologics, including biosimilars.^58^, ^72^ The importance of pharmacovigilance should also be highlighted in medical schools, with the support of national rheumatology societies.^17^ PANLAR is currently drafting a consensus on biosimilars to guide rheumatologists and regulatory authorities when making decisions on biosimilar use and approval.^73^

Our survey of rheumatologists in Latin America aimed to determine the current awareness when prescribing biosimilars and some of the perceived barriers, including nomenclature, automatic substitution, and reporting of possible ADRs because of the use of biosimilars. Results from our survey highlight several issues, including a lack of awareness regarding the availability of biosimilars and automatic substitution. Additionally, nomenclature for biosimilars remains unclear to many rheumatologists, which affects the traceability of biosimilars and, subsequently, the accurate reporting of ADRs associated with their use. Improving the knowledge of rheumatologists on these key issues could facilitate improvements in the pharmacovigilance of biosimilars in Latin America.

The overall goal of pharmacovigilance is to accurately and promptly trace ADRs to a particular product and manufacturer; therefore, agreement on a common definition of, and legislation for, interchangeability of biosimilars is essential for well‐functioning pharmacovigilance systems and has important implications for rheumatologists prescribing these drugs. Clear guidelines on the interchangeability of biologics and biosimilars are needed to ensure patient safety and effective postmarketing pharmacovigilance. Further input from academic bodies and regulatory agencies is vital to establish a common position on these issues.

## ETHICS STATEMENT

The authors state that no ethical approval was needed.

## CONFLICT OF INTEREST

Carlos Pineda and Hugo Sandoval have no potential conflicts of interest to declare. Gilberto Castañeda‐Hernández has received consultancy fees from Amgen, AbbVie, AstraZeneca, Bayer, Boehringer Ingelheim, Eli Lilly, Janssen‐Cilag, Laboratorios Liomont, Laboratorios Sophia, Medix, Merck Serono, Merck Sharp & Dohme, Novartis, Pfizer, Roche, Sanofi, and UCB. Javier Coindreau and Luis Felipe Rodriguez‐Davison are full‐time employees of, and declare stock holdings and/or stock options from, Pfizer Inc.
